# Novel wideband circularly polarized DRA with squint-free radiation characteristics

**DOI:** 10.1038/s41598-021-86381-1

**Published:** 2021-03-30

**Authors:** Mohammad Abedian, Mohsen Khalily, Vikrant Singh, Pei Xiao, Rahim Tafazolli, Ahmed A. Kishk

**Affiliations:** 1 Institute for Communication Systems (ICS), Home of the 5G & 6G Innovation Centres, University of Surrey, Guildford, GU2 7XH UK; 2 Department of Electrical and Computer Engineering, Concordia University, Montreal, QC H3G 2W1 Canada

**Keywords:** Engineering, Electrical and electronic engineering

## Abstract

A new single-fed circularly polarized dielectric resonator antenna (CP-DRA) without beam squint is presented. The DRA comprises an S-shaped dielectric resonator (SDR) with a metalized edge and two rectangular dielectric resonators (RDRs) blocks. Horizontal extension section is applied as an extension of the SDR, and a vertical-section is placed in parallel to the metallic edge. A vertical coaxial probe is used to excite the SDR and the vertical RDR blocks through an S-shaped metal element and a small rectangular metal strip. The two added RDRs that form an L-shaped DR improve the radiation characteristics and compensate for the beam squint errors. A wideband CP performance is achieved due to the excitation of several orthogonal modes such as $TE_{\delta 11}^x$, $TE_{1\delta 1}^y$, $TE_{121}^z$, $TE_{112}^y$, $TE_{131}^x$, and $TE_{311}^y$. The experimental results demonstrate an impedance bandwidth of approximately $66.8\%$ (3.71–7.45 GHz) and a 3-dB axial-ratio (AR) bandwidth of about $54.8\%$ (3.72–6.53 GHz) with a stable broadside beam achieving a measured peak gain of about $4.64 \, {\text{dBic}}$. Furthermore, a 100% correction in beam squint value from $\theta = 41^\circ$ to $\theta = 0^\circ$ with respect to the antenna boresight is achieved.

## Introduction

Dielectric resonator antennas (DRAs) have been widely studied due to their attractive features such as high radiation efficiency, low loss, no surface wave, various excitation mechanisms, light-weight, geometrical flexibility, and compact antenna size^1–3^. The 3D structure of DRAs provides a higher degree of flexibility over microstrip antennas that have been widely used in various systems^4,5^ but suffer from limited bandwidth and low radiation efficiency caused by the conduction losses^6^. Among the three main DRA shapes, rectangular DRA (RDRA) offers an added advantage of higher design flexibility as its three-dimensional structure can have different aspect ratios^7^.

In addition, the amalgamation of various DRA shapes and coupling schemes provides the flexibility to obtain the desired linear or circular polarization (CP). Linear polarization (LP) is sensitive to multipath reception and misalignment between transmitting and receiving antennas. In contrast, CP waves, which are usually excited by two orthogonal linearly polarized waves of equal amplitude and 90-degree phase difference, have received much attention because they offer more flexibility for the transmitter and receiver orientations along with the capability of mitigating polarization mismatch and suppressing multipath interference^8–11^. Recently, different shapes of the DR and various feeding mechanisms have been introduced to achieve wideband DRAs, operating in CP, as a pathway to fulfill spectrum requirements by taking the advantages of better mobility and less multipath effects^12–25^. Two orthogonal modes must be excited with a quadratic phase difference to design circularly polarized DRA, which can be realized by single or multiple feed mechanisms. The single feed mechanism has a relatively simple structure but limited CP bandwidth. Moreover, the antenna’s radiation performance can be degraded due to the asymmetry of the excitation. On the other hand, having a multiple feed network provides a wider CP bandwidth but results in a large and complex feeding structure. In the literature, several single-point feed DRAs have been introduced to enhance the CP bandwidth^15–25^. For instance, in^17^, a wideband CP quadruple-strip-fed cylindrical DRA with a CP bandwidth of $25.9\%$ using a pair of $90^\circ$ hybrid couplers has been presented. A trapezoidal DRA excited by an inclined slot has been proposed in^18^, which offers a CP bandwidth of 21.5%. In^19^, a DRA with diagonal slits has been reported, which shows 43% AR bandwidth with an overlapped matching bandwidth of about 36%. Zou et al.^20^ have introduced a RDRA excited by a spiral strip, achieving a CP bandwidth of 25.5%. In parallel, by exciting a RDRA through a unique conformal H-shaped metal strip, a wideband CP DRA with a CP bandwidth of 20% has been achieved^21^. Moreover, Yang et al.^22^ proposed a square DRA excited by a microstrip coupled cross-slot with four vertical metal plates around the DRA, achieving a CP bandwidth of 46.9% with an average gain of $4.69 \, {\text{dBic}}$ within the desired operating band. A simple shaped RDRA with 20.8% AR bandwidth and high radiation efficiency of more than 97% has been presented in^23^. A DRA containing rectangular and two half-split cylindrical DRs excited by a stair-shaped slot has been proposed in^24^ which offers 41.01% AR bandwidth but low gain. A hybrid antenna with a cylindrical DR and dual vertical microstrip lines arranged perpendicularly for obtaining wideband CP bandwidth of 24.6% has been reported in^25^. However, it is observed that the radiation bandwidth is narrower than the matching bandwidth as the radiation characteristics changes within the desired bandwidth.

On the other hand, for a CP antenna, the antenna beam steering performance can be adversely affected by the beam squint phenomenon and reducing antenna broadside gain. Beam squint is caused by exciting higher-order modes in the presence of the desired mode due to the asymmetry of the excitation and the geometry. Such squint is undesirable for communication as the co-component’s peak amplitude is not in the broadside direction. This behavior introduces frequency selectivity to the wideband signal, resulting in the CP antenna’s poor performance and compromised system efficiency. Therefore, it poses a significant limitation on its many practical applications, such as satellite and polarimetry^26^. One of the common techniques to excite orthogonal modes is to design an asymmetric resonator, but it may shift the main beam towards different angles rather than boresight. Furthermore, to widen the CP, the position of feeding structure with respect to the resonator plays an important role and can cause the main beam’s deviation from boresight. For example, the CP-DRAs in^19,21,23,27–29^ suffered from a large squint angle, which is more than 20$^{\circ }$ due to the excited orthogonal modes’ asymmetry in magnitude and phase between them. This article has proposed a new technique to remove the beam squint effect and widen the CP bandwidth with radiation characteristic enhancement. The design procedure and the measured and simulated results are discussed in the following sections.

## Antenna configuration and physical working mechanism

**Figure 1:**
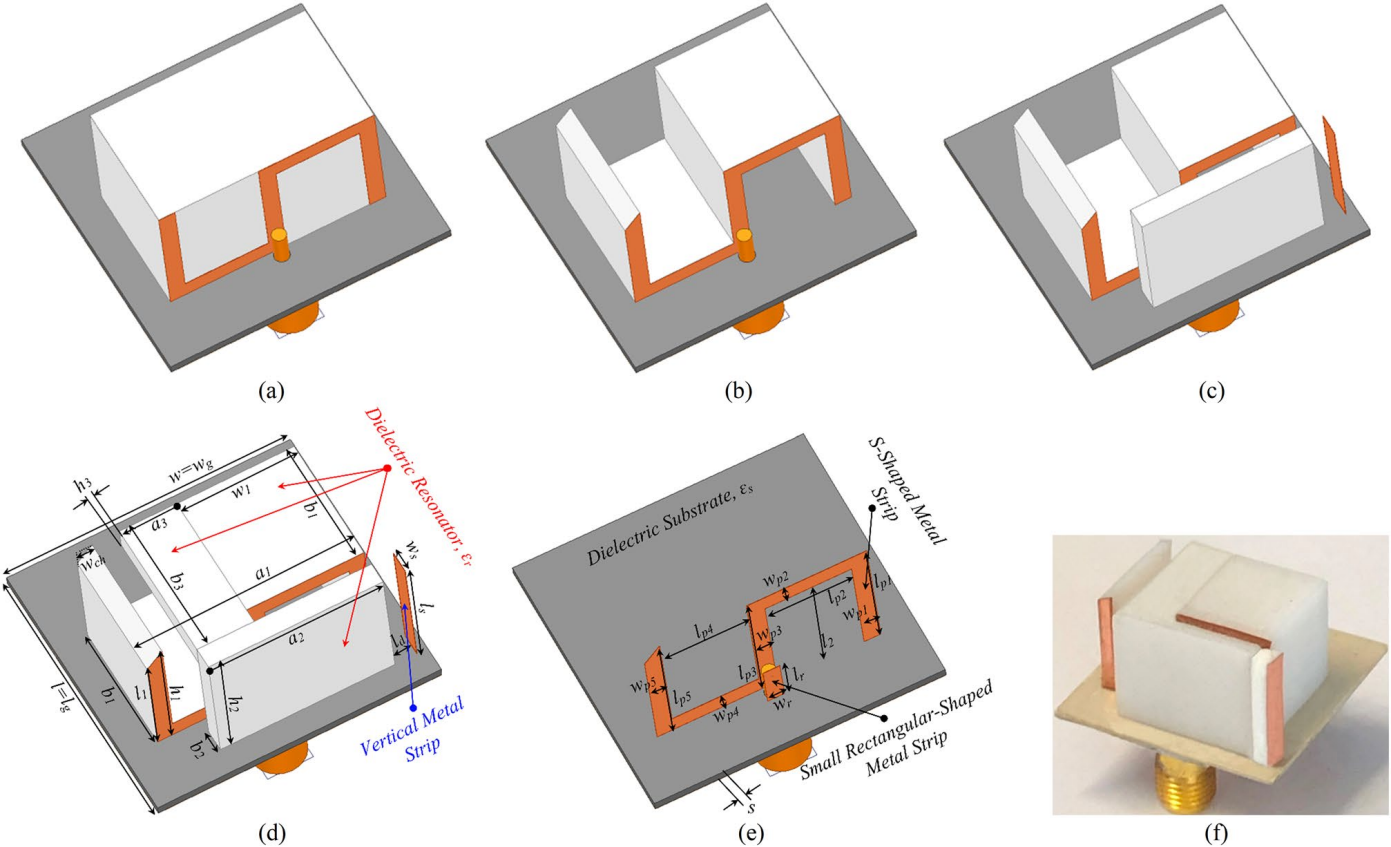
Evolution of the proposed CP DRA: (**a**) RDRA, (**b**) SDRA (Antenna I), (**c**) SDRA with the first added RDR and vertical metal strip (Antenna II), (**d**) SDRA with the first and the second added RDRs (Antenna III); Geometry of the proposed CP DRA, (**e**) feeding mechanism, and (**f**) the prototype of proposed DRA. ($l = l_g = 26$, $w = w_g = 26$, $l_{p1} = l_{p3} = l_{p5} = 12$, $l_{p2} = l_{p4} = 7.75$, $w_{p1} = w_{p3} = w_{p5} = 1.5$, $w_{p2} = w_{p4} = 2$, $l_r = 4$, $w_r = 2$, $l_s = 12$, $w_s = 2$, $l_d = 2$, $w_{ch} = 1.5$, $l_1 = 10.5$, $l_2 = 10$, $w_1 = 10.75$, $a_1 = 20$,$b_1 = 12$, $h_1 = 12$, $a_2 = 16$, $b_2 = 2$, $h_2 = 12$, $a_3 = 5.25$, $b_3 = 13.345$, $h_3 = 2$, $\varepsilon _r = 10$, $\varepsilon _s = 3$, $= 0.75$. Unit: *mm*).

The evolution and schematic of the proposed wideband CP-DRA is demonstrated in Fig. 1. Figure 1a represents the proposed DRA’s initial design, where the CP bandwidth is limited to $$4\%$$. Hence, the RDR is modified to enhance the CP bandwidth by introducing two equal hollow blocks in RDR with the key parameters $$l_{p2} = l_{p4}$$ and $$l_2$$ to form an SDR (see Fig. 1b) to reduce DR’s Q-factor and enhance impedance matching. Figure 1b–d illustrate three different geometries of the DRA denoted as Antenna I, Antenna II, and Antenna III, respectively. Fig. 1d depicts the final design of proposed DRA, indicating the design parameters, while Fig. 1e presents the parameters associated with the feeding mechanism. To achieve wideband CP, an SDR with dimensions $$a_1$$ $$\times$$ $$b_1$$ $$\times$$ $$h_1$$ excited by a coaxial probe through an S-shaped metal strip $$l_p$$ $$\times$$ $$w_p$$ (denoted as Antenna I, see Fig. 1b). The proposed DRA is supported by a grounded $$26 \; (x{\text{-}}axis) \, \text{$mm$} \times 26 \; (y{\text{-}}axis) \, \text{$mm$}$$ Rogers RO3003 substrate with a permittivity of $$\varepsilon _s$$ = 3 and a thickness of *s* = $$0.75 \, \text{$mm$}$$. Secondly, a RDR with dimensions of $$a_2$$ $$\times$$ $$b_2$$ $$\times$$ $$h_2$$ is placed close to the SDR, excited by a small rectangular-shaped metal, $$l_r$$ $$\times$$ $$w_r$$, attached to the coaxial probe. In addition, a vertical metal strip with dimensions of $$l_s$$ $$\times$$ $$w_s$$ is placed on one side of the new resonator at a distance of $$l_d$$, resulting in a wider antenna bandwidth (denoted as Antenna II, see Fig. 1c). Finally, another RDR of volume $$a_3$$ $$\times$$ $$b_3$$ $$\times$$ $$h_3$$ is attached to the aforementioned DRs to improve the radiation characteristics (denoted as Antenna III, see Fig. 1d). All the DRs are made of ECCOSTOCK HiK dielectric material with relative permittivity $$\varepsilon _r$$ = 10 and loss tangent $$\tan \, \delta$$ = 0.002.

In this work, CST Microwave Studio 2019 is used to analyze and optimize the proposed antenna. From a topological point of view, this work’s main contribution is exciting a single-fed CP antenna by applying an S-shaped parasitic strip attached to the DR block and, at the same time, compensating the beam squint error. It is worth noting that for further CP bandwidth improvement, two equal hollow blocks are introduced in the RDR. In parallel, two RDR blocks are placed to overcome the beam squint issue. This section describes the design process of the proposed DRA and physical mechanisms.

### Wideband CP-DRA design

As illustrated in Fig. 1a, to couple the energy to the RDRA and excite multiple resonances in close vicinity, an S-shaped strip connected to the coaxial probe is attached to the RDRA while the initial dimensions of the RDRA are calculated using the dielectric waveguide model (DWM) equations^2^. Here, to generate CP fields, two orthogonal $TE^x$ and $TE^y$ modes with equal amplitude and $90^\circ$ phase difference are excited by using an S-shaped metal strip to indicate right-hand CP (RHCP) and left-hand CP (LHCP) waves components as follows^8^:

1 $$\begin{aligned} E_{RHCP}= & {} \frac{1}{\sqrt{2}} \; (E_x + j E_y), \end{aligned}$$

2 $$\begin{aligned} E_{LHCP}= & {} \frac{1}{\sqrt{2}} \; (E_x - j E_y). \end{aligned}$$

According to image theory, the S-shaped strip current produces two distinct modes, common and differential, which correspond to exciting the $TE^x$ and $TE^y$ modes, respectively. The corresponding field components of each mode ($TE^x$ and $TE^y$) are equal and can be expressed as follows^7^:

3 $$\begin{aligned} TE^x (E_y) = TE^y (E_x) = - AK_z \;\cos ({K_x} x)\;\cos ({K_y} y)\;\sin ({K_z} z). \end{aligned}$$

**Figure 2:**
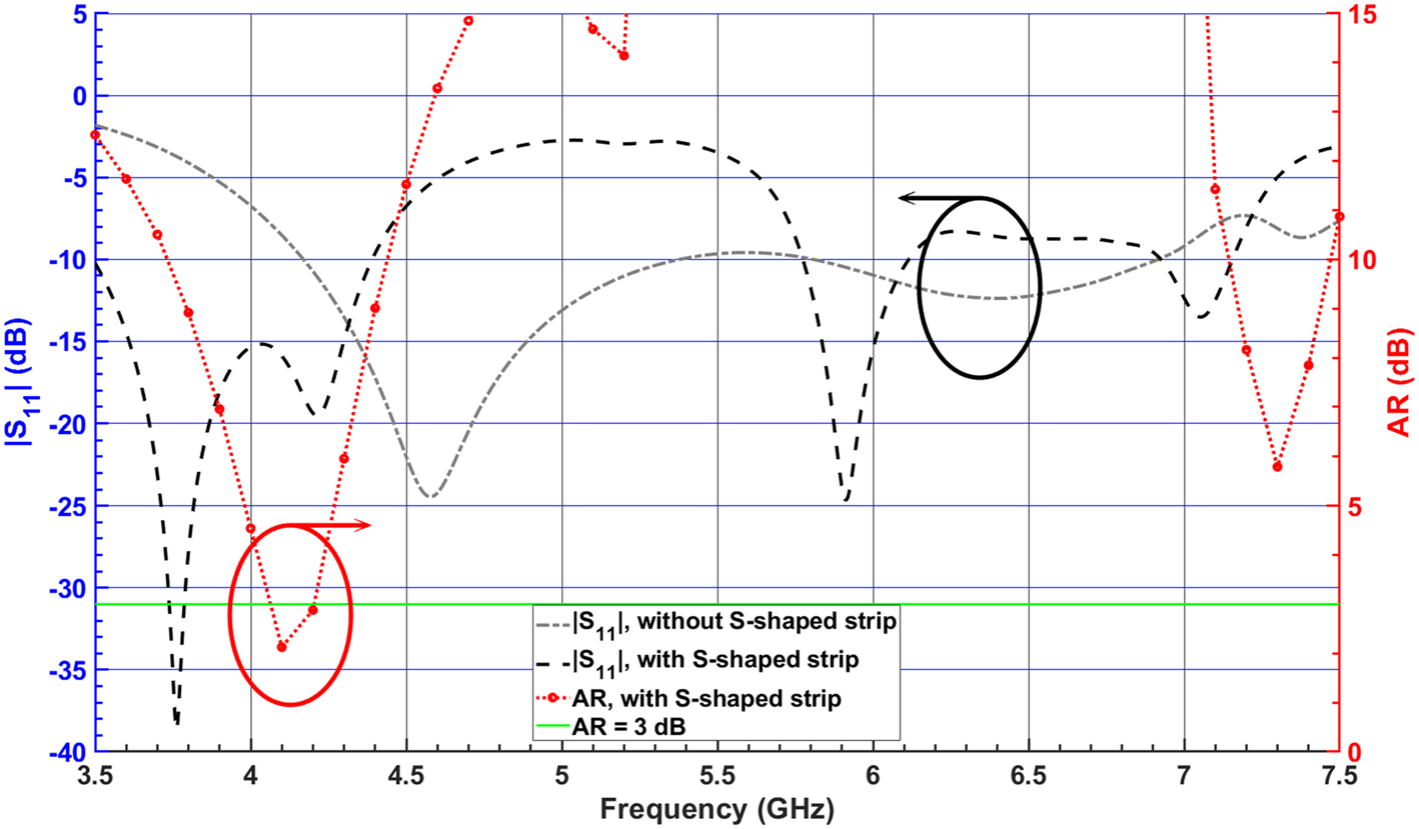
Simulated $|S_{11}|$ and AR of the RDRA with and without an S-shaped metal strip.

**Figure 3:**
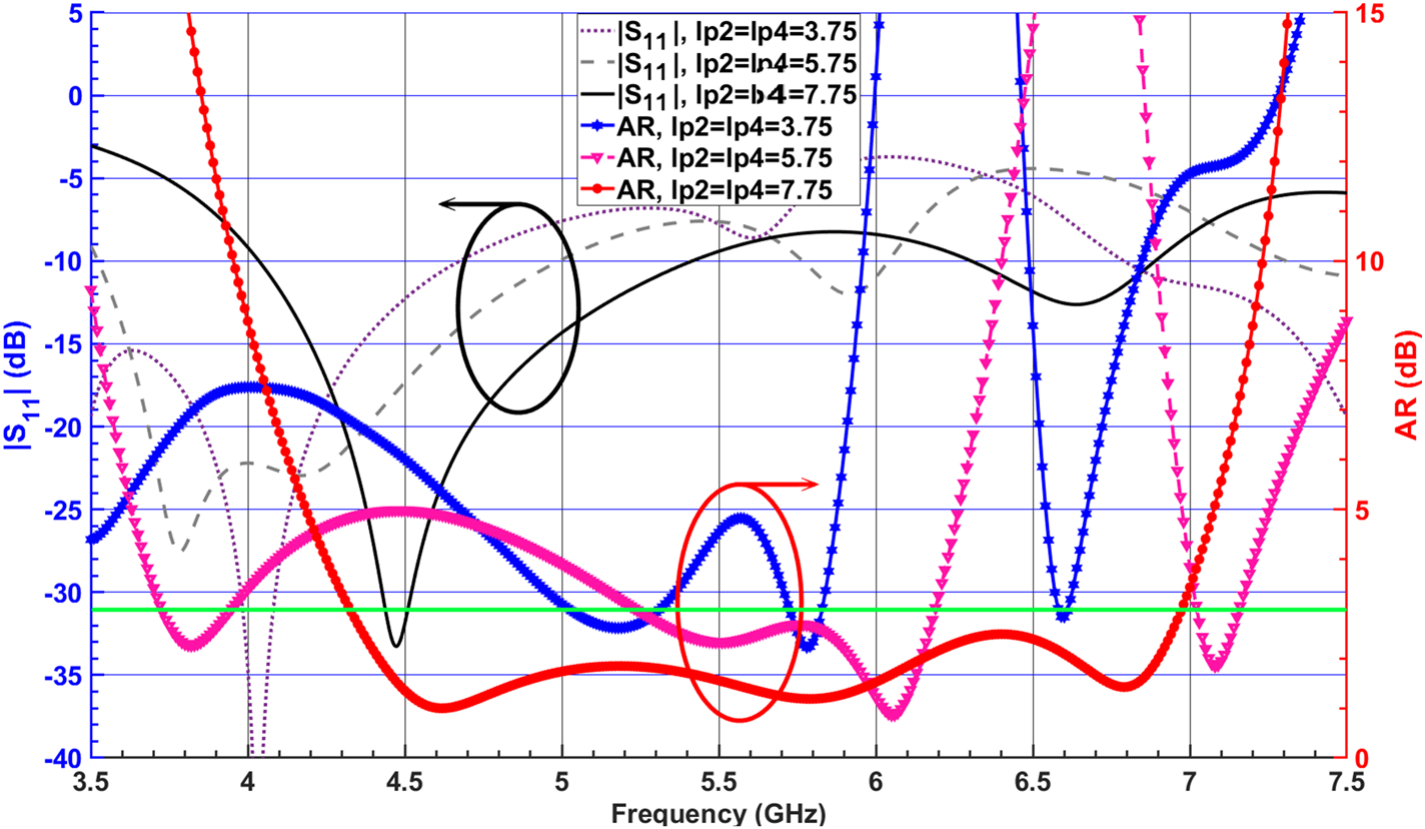
Simulated $|S_{11}|$ and ARs of the SDRA with different values of $l_{p2} = l_{p4}$.

Figure 2 shows the simulated $|S_{11}|$ and AR of the RDRA with and without an S-shaped parasitic element. It can be clearly observed that the S-shaped strip provides wider impedance bandwidth with an overlapping broadside AR bandwidth around 4.1 GHz. In order to obtain a wider CP bandwidth, the RDRA is modified to form an SDRA. Figure 3 shows the AR graph versus different values of $l_{p2} = l_{p4}$, at the resonance frequency of the corresponding first and second orthogonal mode pairs. It can be seen that as the length of $l_{p2} = l_{p4}$ increases, 3-dB AR bandwidth improves for the first and second orthogonal modes.

**Figure 4:**
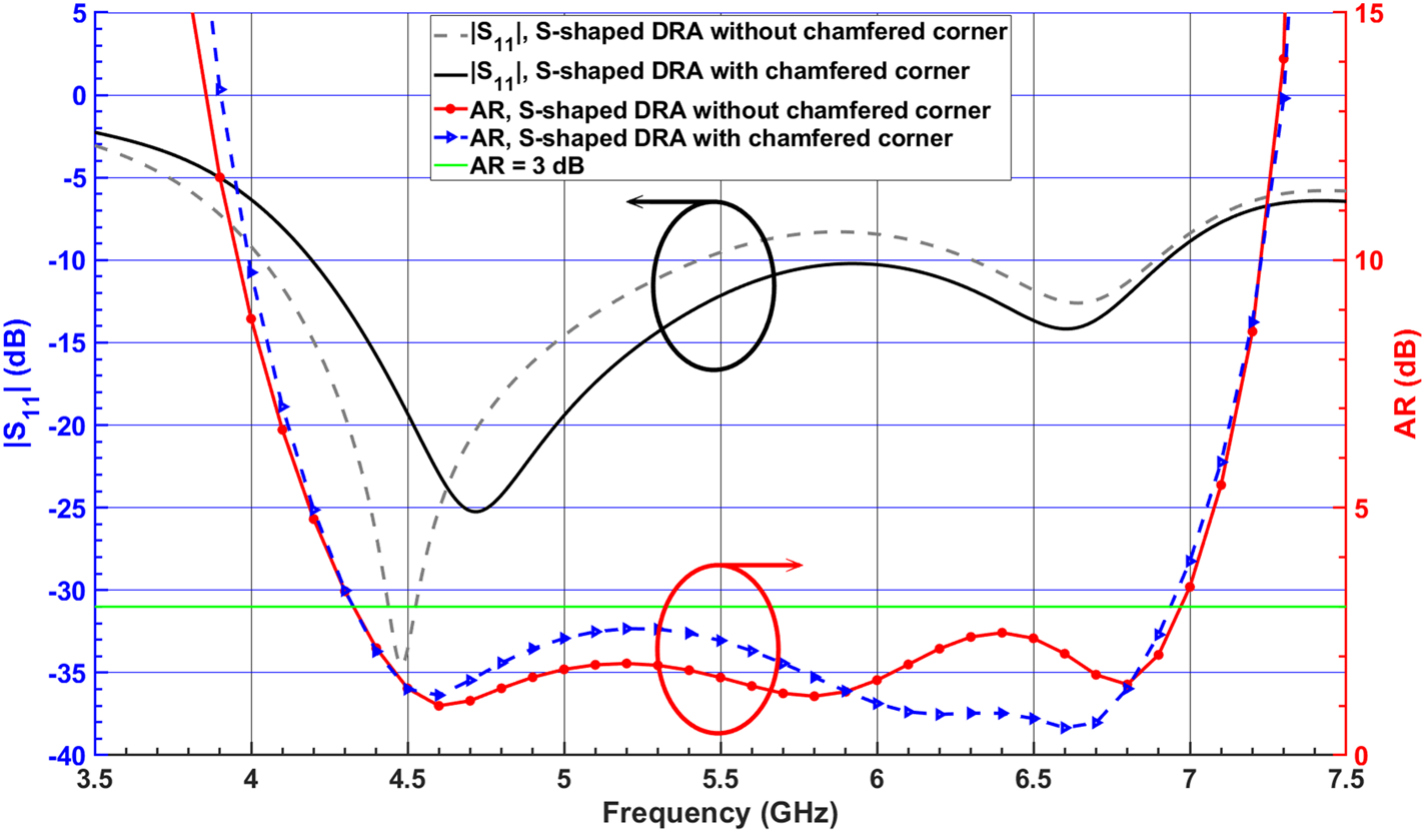
Simulated $|S_{11}|$ and ARs of the SDRA with and without chamfered corner.

**Figure 5:**
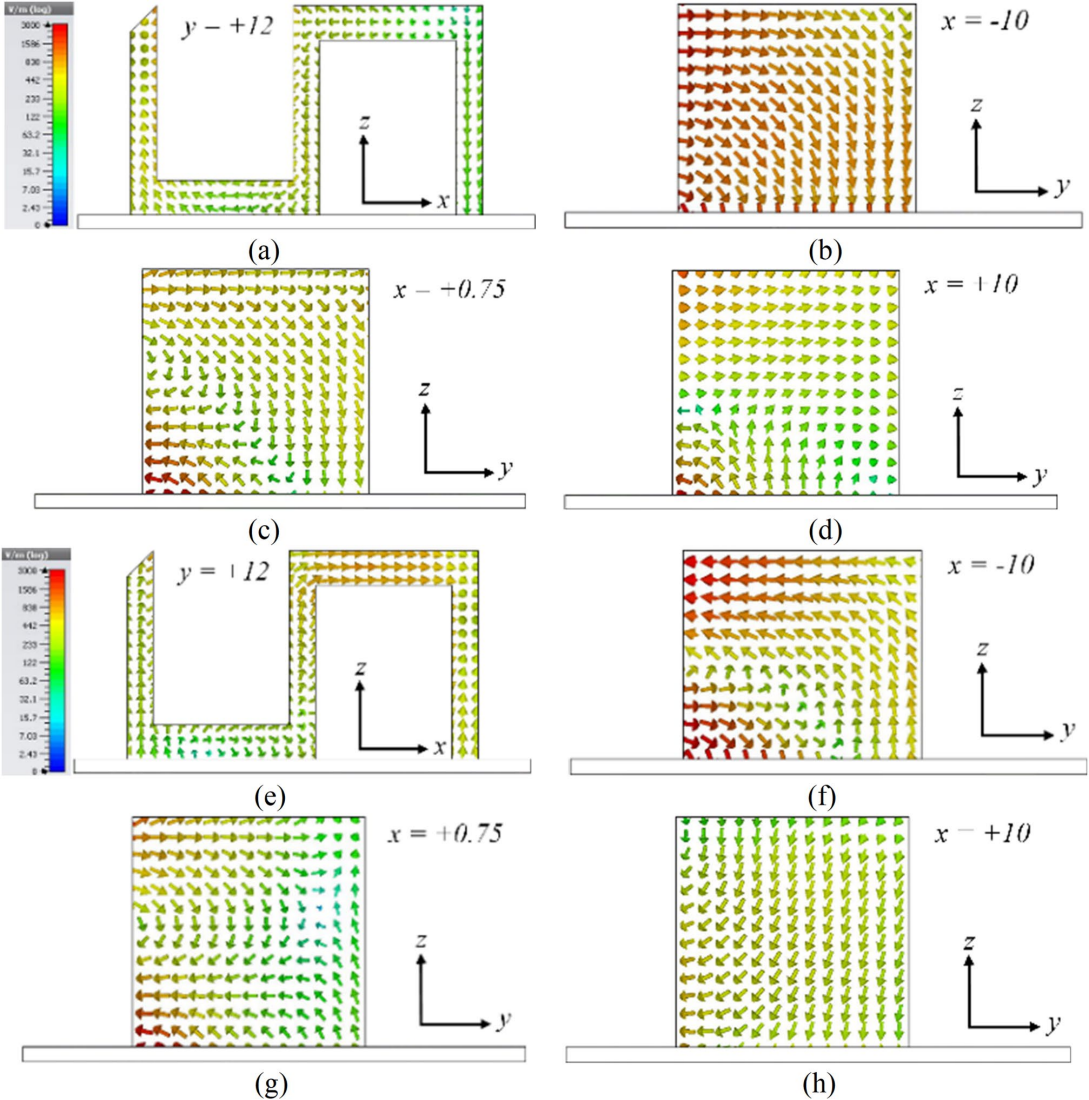
Simulated electric field vectors on different surfaces of the SDR; at 4.6 GHz (**a**) $$TE_{1\delta 1}^y$$$$\measuredangle$$ $$45^\circ$$ ($$y = 12 \, {\text{mm}}$$), (**b**) $$TE_{\delta 11}^x$$$$\measuredangle$$ $$135^\circ$$ ($$x = -\,10$$), (**c**) $$TE_{\delta 11}^x$$$$\measuredangle$$ $$135^\circ$$ ($$x = +\,0.75$$), and (**d**) $$TE_{\delta 11}^x$$$$\measuredangle$$ $$135^\circ$$ ($$x = +\,10$$); at 6.5 GHz (**e**) $$TE_{311}^y$$$$\measuredangle$$ $$45^\circ$$ ($$y = 12 \, \text{{mm}}$$), (**f**) $$TE_{131}^x$$$$\measuredangle$$ $$135^\circ$$ ($$x = -\,10$$), (**g**) $$TE_{131}^x$$$$\measuredangle$$ $$135^\circ$$ ($$x = +\,0.75$$), and (**h**) $$TE_{131}^x$$$$\measuredangle$$ $$135^\circ$$ ($$x = +\,10$$); (color bar shows the amplitude of the E-field.

The SDRA provides a wider 3-dB AR bandwidth, as shown in Fig. 4. However, the corresponding impedance bandwidth shows a degradation and does not satisfy the − 10-dB $|S_{11}|$ around 6 GHz. To improve the impedance matching, a corner of the SDRA is chamfered with $45^\circ$, called Antenna I, as shown in Fig. 1b. The simulated E-field distributions at 4.6 GHz and 6.5 GHz are shown in Fig. 5 for Antenna I. The figure indicates that orthogonal modes with equal magnitude and quadrature-phase are excited at 4.6 GHz and 6.5 GHz. It is worth mentioning that the resonant modes excited across the desired CP band resemble orthogonal $TE_{\delta 11}^x$, $TE_{1\delta 1}^y$, $TE_{131}^x$, and $TE_{311}^y$ modes.

### Radiation characteristic improvement and beam squint reduction

**Figure 6:**
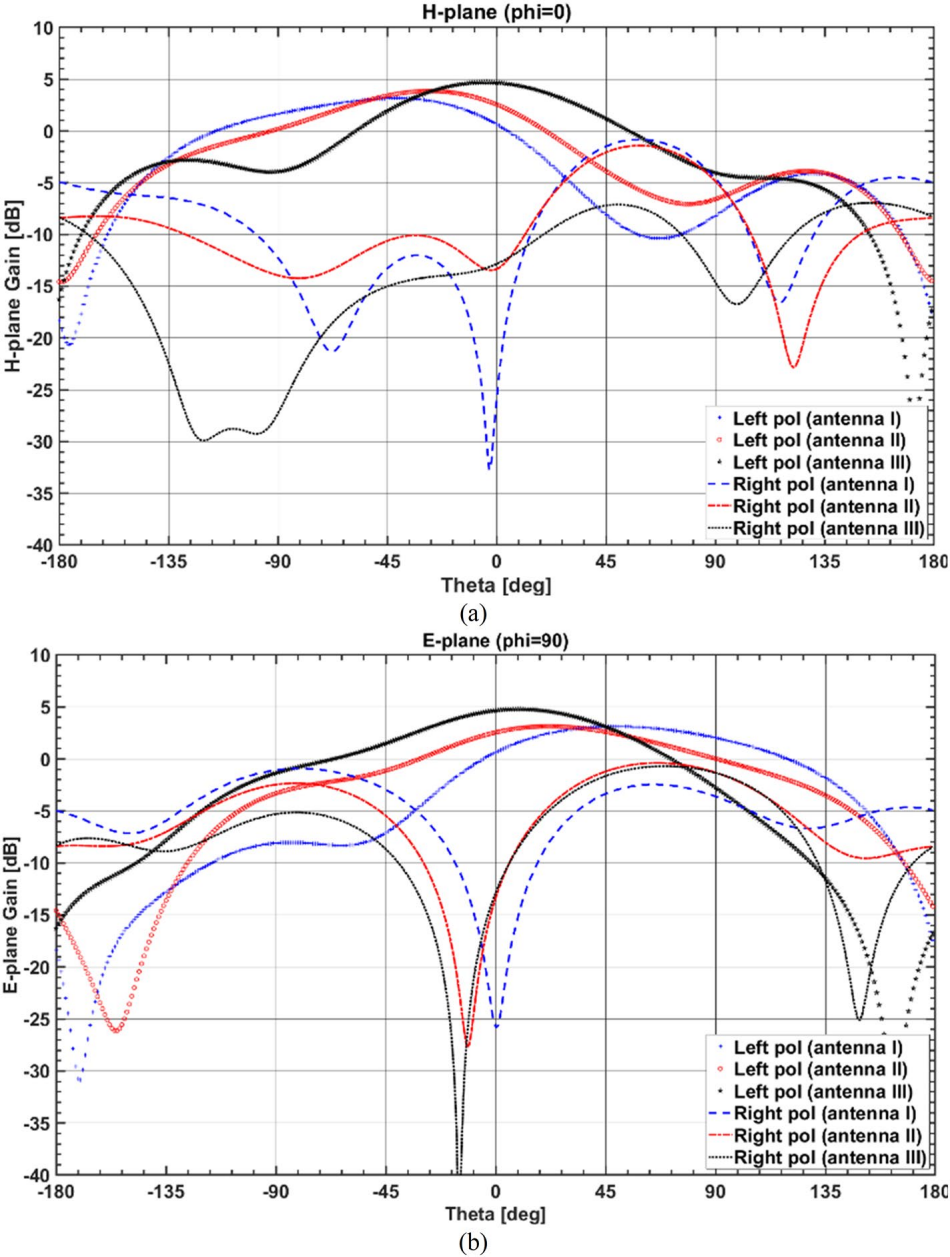
Simulated (**a**) H-plane and (**b**) E-plane radiation patterns of Antenna I, Antenna II, and Antenna III at 6.5 GHz.

Figure 6 illustrates the simulated H- and E-plane radiation patterns at 6.5 GHz for three different cases: Antenna I, Antenna II, and Antenna III, as shown in Fig. 1b–d, respectively. As for Antenna I, it is observed that the radiation pattern in the broadside direction deviates from the $+\,z$-direction with a high beam squinting of $-\,41^\circ$, which does not satisfy the required specifications for CP antennas.

**Figure 7:**
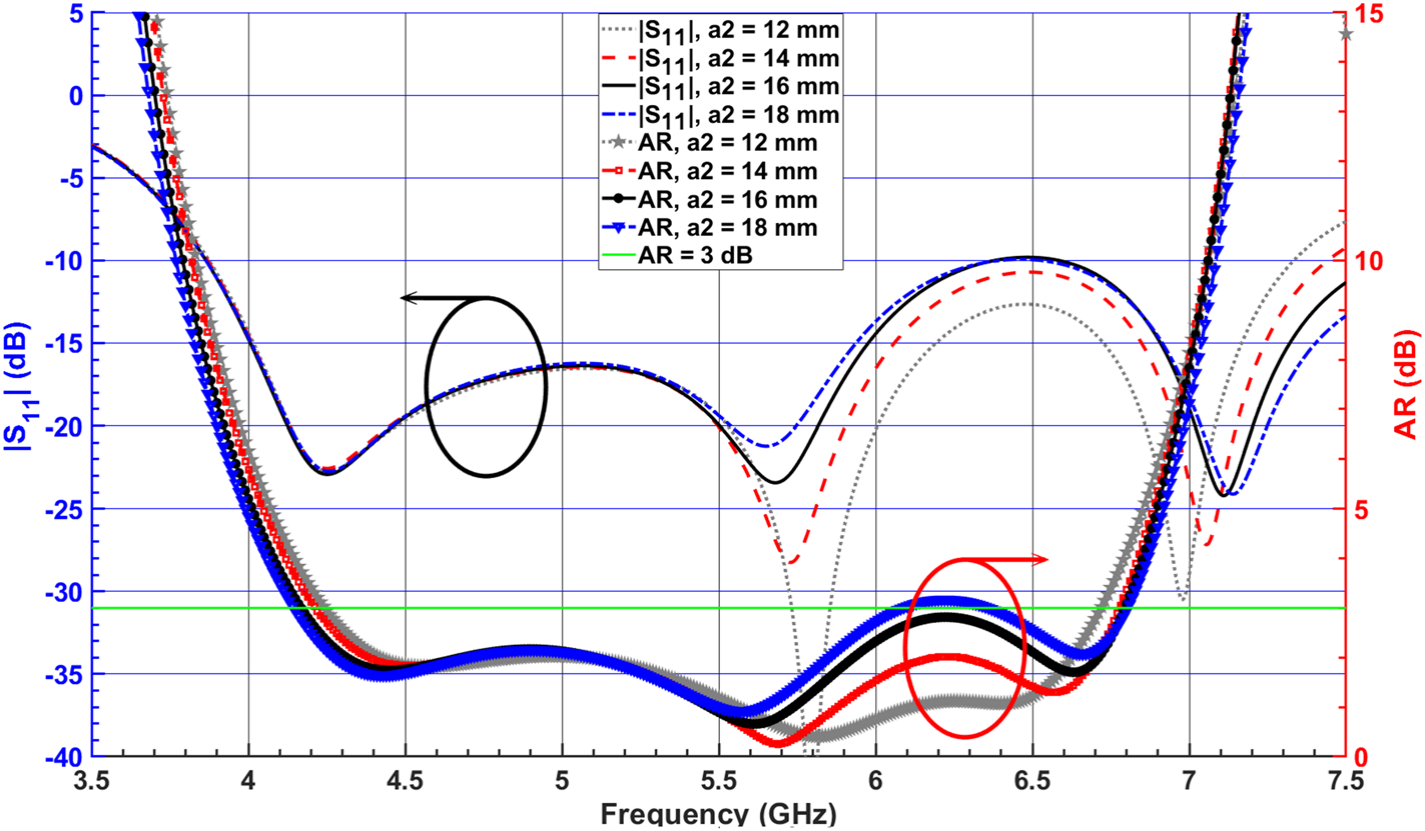
Simulated $|S_{11}|$ and ARs for the DRA (Antenna II) versus various total lengths $a_2$ of the first added RD.

To fix the beam squint effect at 6.5 GHz, a RDR is placed in front of the SDRA to concentrate radiative fields toward the boresight (see Fig. 1c). The RDR is excited by a small rectangular-shaped parasitic strip attached to the coaxial probe to improve the symmetry of the radiation patterns at the upper bands due to exciting higher-order modes inside the RDR. Another important parameter that has a significant effect on the AR and $|S_{11}|$ is $a_2$. Figure 7 illustrates the effect of varying $a_2$ on the AR and $|S_{11}|$, indicating that by increasing $a_2$, from 10 to 18 mm, the higher edge of the bandwidth is increased. The separation between the corresponding resonant frequencies of the excited orthogonal modes inside the DRA increases the AR bandwidth. It is noticed that the beam squinting is reduced to $28^\circ$, which leads to an increased LHCP gain from $3.17 \, {\text{dBic}}$ to $3.8 \, {\text{dBic}}$ at 6.5 GHz.

**Figure 8:**
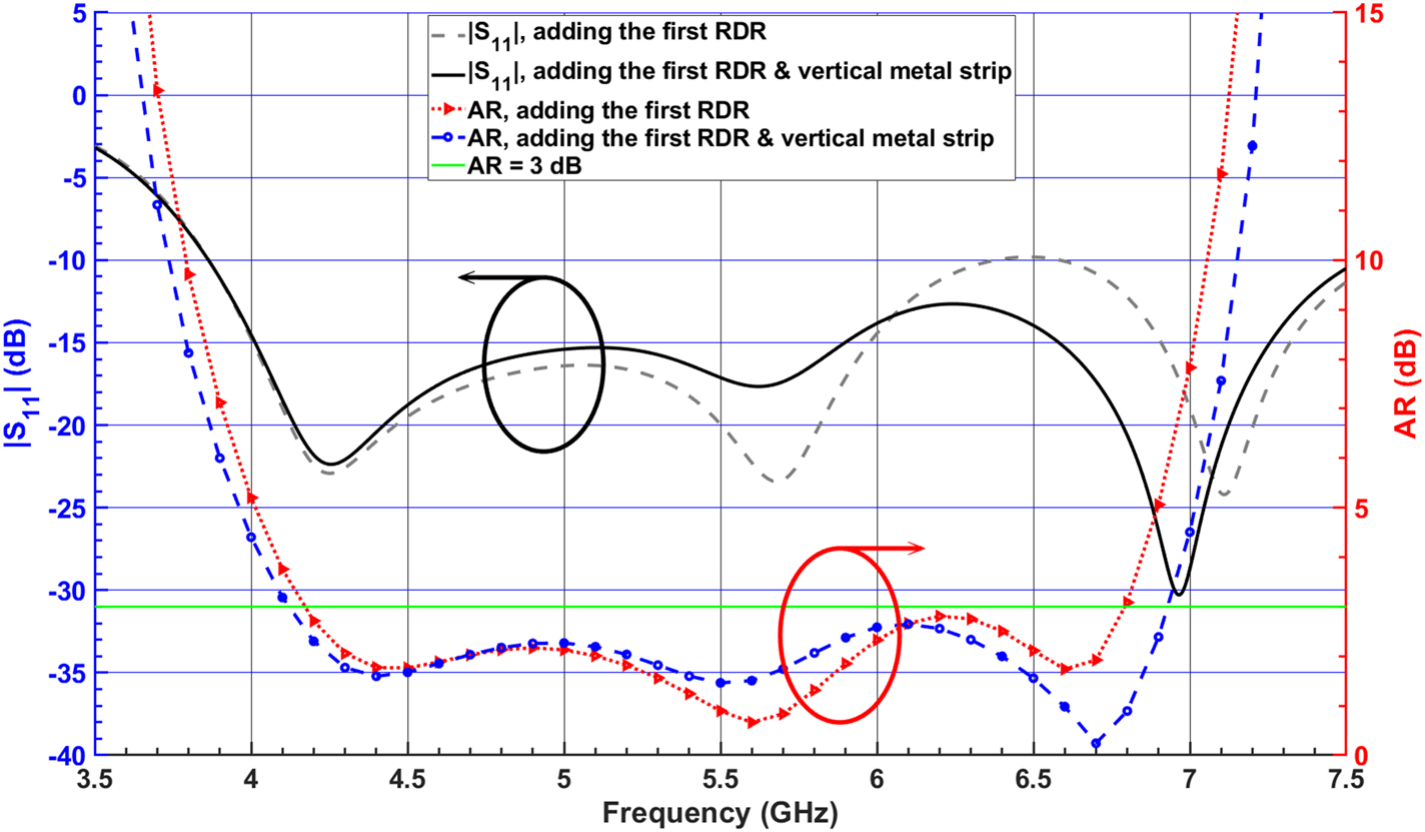
Simulated $|S_{11}|$ and ARs of the DRA (Antenna II), adding the first RDR block and vertical metal strip.

A vertical metal strip is then placed on one side of the thin RDR slab at a distance of $$l_d = 2 \, \text{$mm$}$$, as illustrated in Fig. 1c. By applying the vertical metal strip, the CP bandwidth and impedance matching are enhanced because of the increased effective resonator dimension in the x-direction. This leads to a downward shift in the resonant frequency of the corresponding higher-order modes^30^, as shown in Fig. 8.

**Figure 9:**
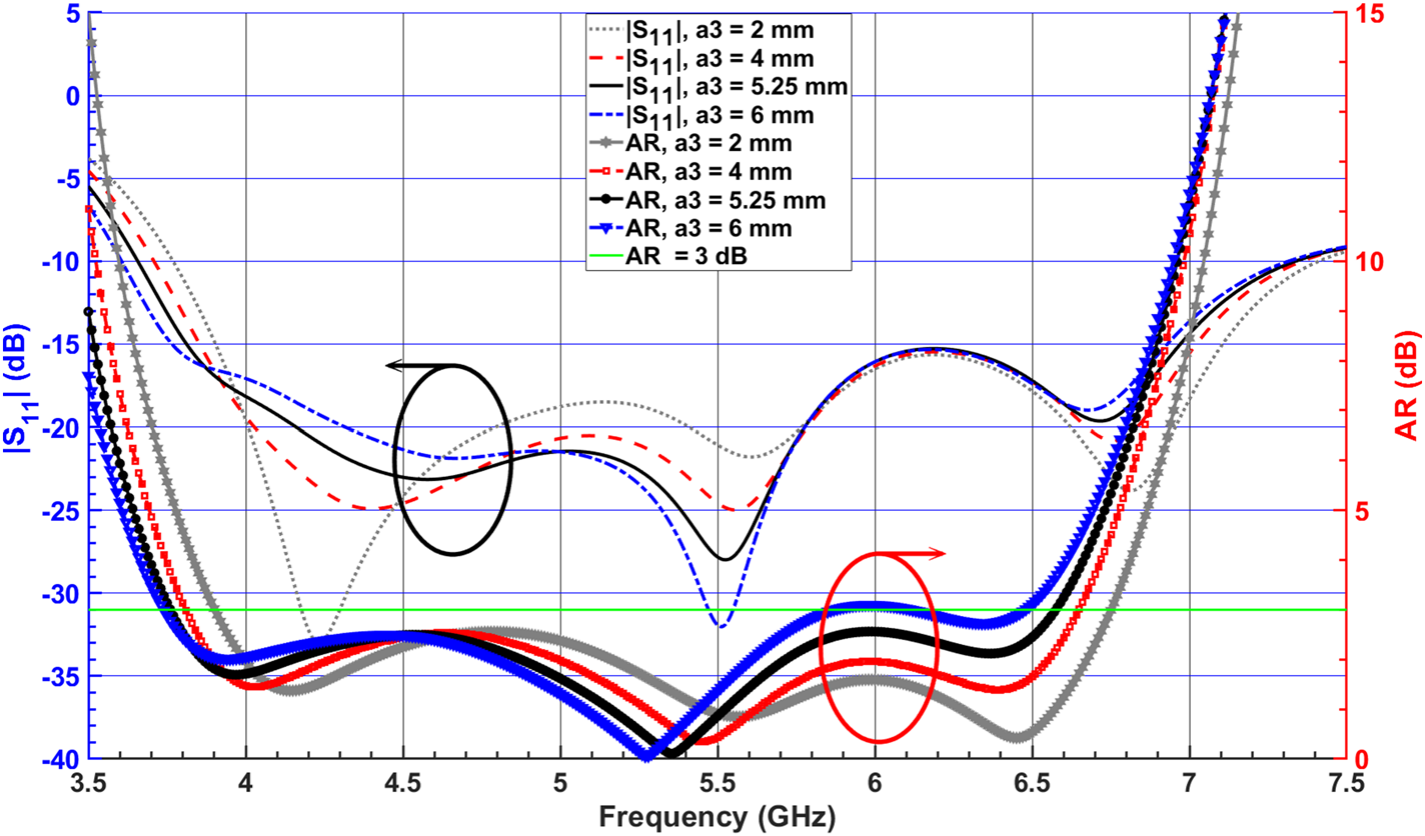
Simulated $|S_{11}|$ and ARs for the DRA (Antenna III) with various total widths $a_3$ of the second added RDR.

**Figure 10:**
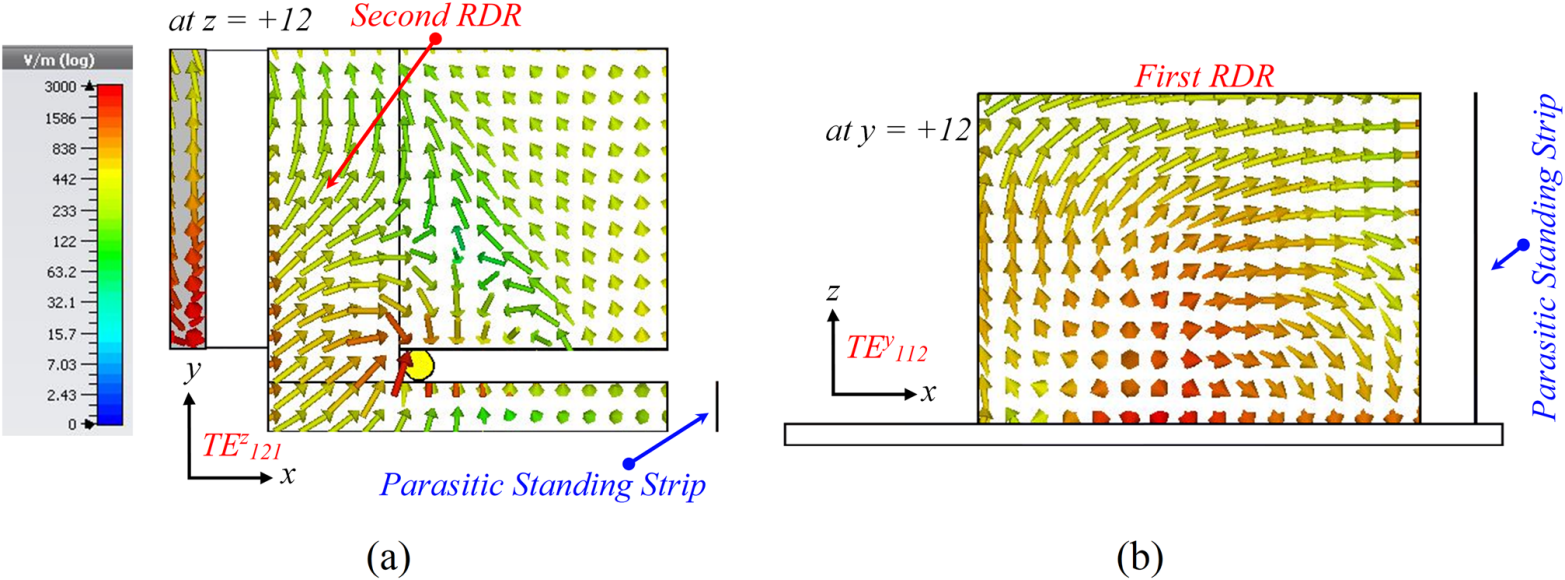
Simulated electric field vectors on the top surface of the SDR and the vertical surface of the first RDR at 5.5 GHz (**a**) *xy*-plane ($$TE_{121}^z$$) $$\measuredangle$$ $$0^\circ$$ ($$z = 12 \, \text{$mm$}$$), (**b**) *xz*-plane ($$TE_{112}^y$$) $$\measuredangle$$ $$90^\circ$$ ($$y = 12 \, \text{$mm$}$$); (color bar shows the amplitude of the E-field).

Finally, to further minimize the beam squint, the second RDR is horizontally attached to the aforementioned DR blocks in the y-direction, as shown in Fig. 1d. The second RDR effect with width $a_3$, on the antenna performance, is exhibited in Fig. 9. It is observed that the AR and $|S_{11}|$ are shifted down to the lower frequency as the width of the second RDR increases from 2 to 6 mm. The widest overlapping bandwidth is achieved at $5.25 \, mm$. Referring to Fig. 6, the boresight gain increases from $3.8 \, {\text{dBic}}$ at $\theta = -\,28^\circ$ to $4.7 \, {\text{dBic}}$ at $\theta = 0^\circ$ for $6.5 \,$ GHz frequency. It is noted that by applying the horizontal RDR, $TE_{121}^z$ mode is excited inside the SDR, which is orthogonal to the existing $TE_{112}^y$ mode excited within the first RDR, as shown in Fig. 10. Furthermore, by increasing the effective resonator dimension in the y-direction, the total ratio of dominant orthogonal modes inside the DRA can be improved. This results in pattern rotation towards $\theta = 0^\circ$ and gain enhancement. Based on this phenomenon, the 3-dB AR and impedance bandwidths shift down to the lower band, resulting in a wider impedance bandwidth of about 66.9% (3.66–7.34 GHz) and 3-dB axial-ratio (AR) bandwidth of about 54.54% (3.76–6.58 GHz).

## Measured and simulated results

In this work, to reduce the complexity of design and fabrication difficulty, two additional DR blocks are combined to form an L-shaped DR. Therefore, one S-shaped and L-shaped DR blocks are placed and fixed in the desired location to achieve satisfactory performance. In addition, a vertical metal strip is glued on a ROHACELL HF Foam ($\varepsilon _{ro}$ = 1.04), and then the L-shaped DR and foam are assembled on top of the substrate using RTV silicone adhesive ($\varepsilon _g \approx 3$) to construct the CP DRA with a vertical metal strip. There are various techniques to measure the axial ratio^8,9^. In the proposed work, the right-hand CP (RHCP) and left-hand CP (LHCP) patterns, and AR are measured using the method proposed in^8^. The dielectric resonator antenna radiation patterns are measured by rotating a linearly polarized horn antenna as a transmitter considering two planes $\phi = 0^\circ and \; 90^\circ$. Meanwhile, the AR is defined using a spinning source antenna with linear polarization in the experimental setup, which requires the source horn to rotate around its z-axis in the ($\phi$-direction) while moving the antenna under test (AUT) in the azimuth direction.

### S-parameter and axial ratio measurement

**Figure 11:**
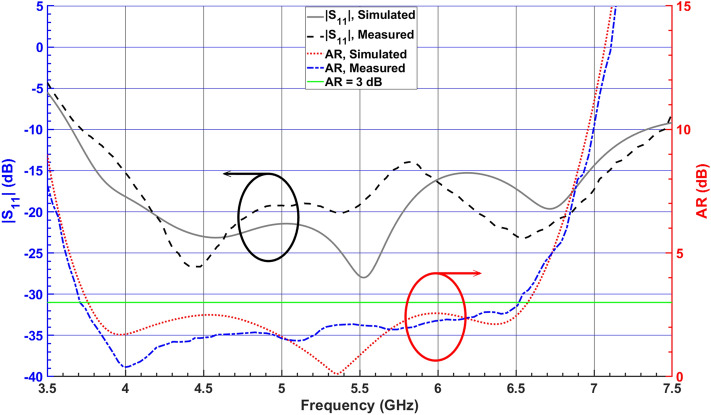
Simulated and measured $|S_{11}|$ and ARs of the proposed CP DRA.

The simulated and measured frequency response of 3-dB AR and $|S_{11}|$ of the proposed antenna is depicted in Fig. 11, representing a close agreement between the simulated and measured results. The CP-DRA provides an impedance bandwidth of about 66.8% (3.71–7.45 GHz) and a 3-dB axial-ratio bandwidth of about 54.8% (3.72–6.53 GHz).

### Far-field measurement

**Figure 12:**
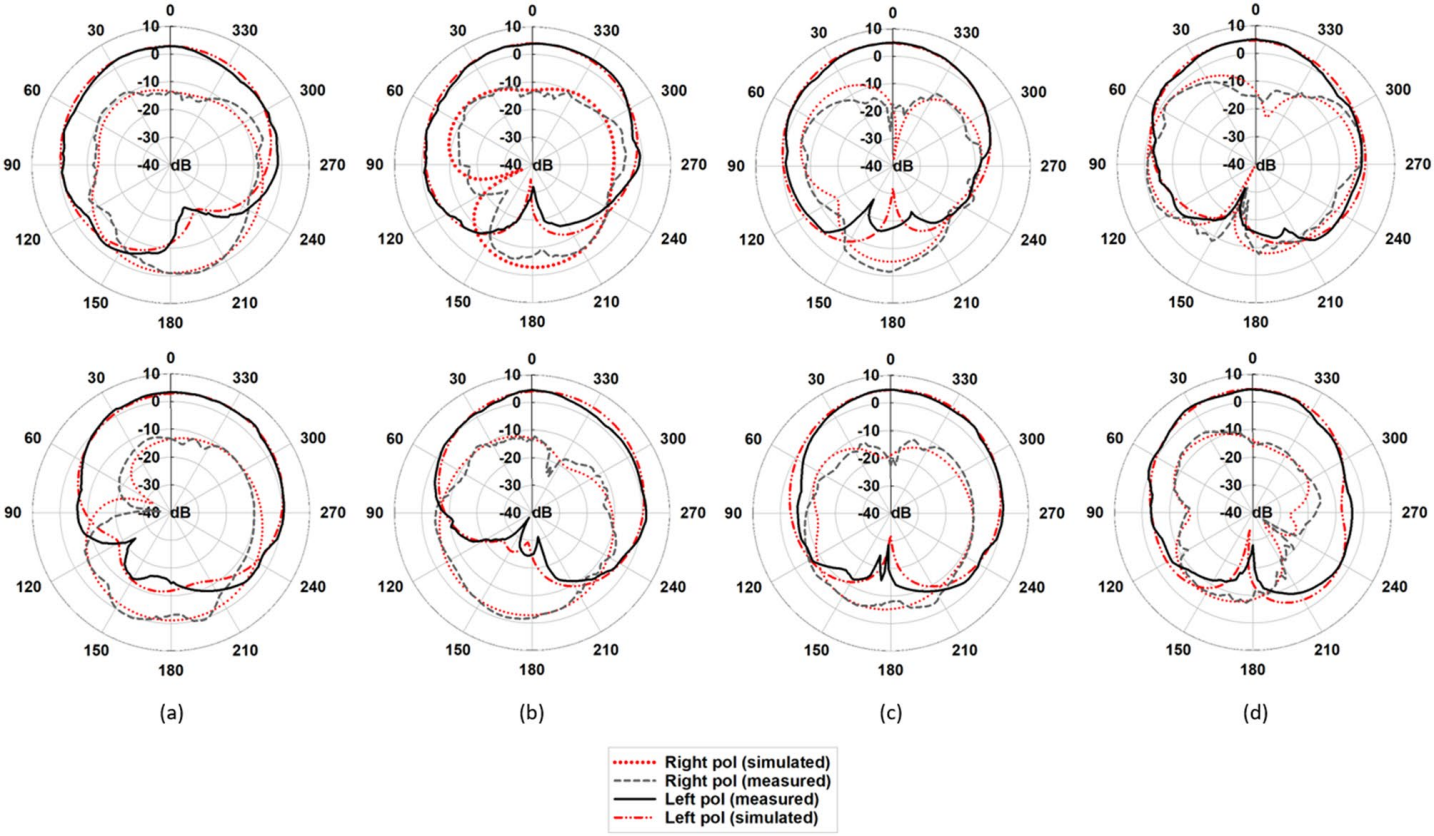
Simulated and measured LHCP and RHCP radiation patterns in *yz*-plane (up) and *xz*-plane (down) at (**a**) 3.76 GHz, (**b**) 4.6 GHz, (**c**) 5.5 GHz, and (**d**) 6.5 GHz. Red and black curves represent simulated and measured results, respectively.

**Figure 13:**
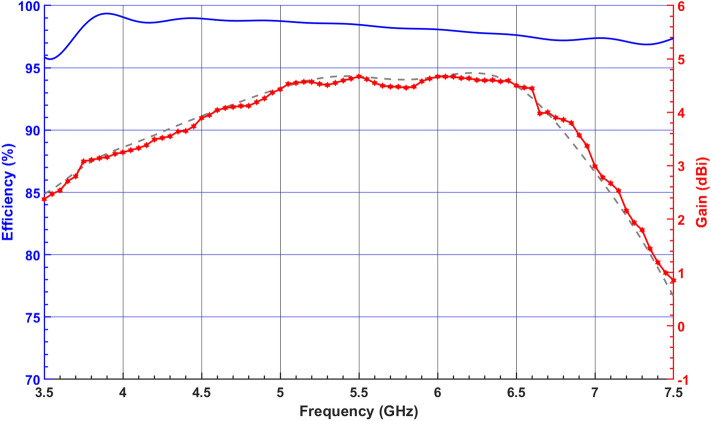
Measured and simulated boresight gain of the proposed CP-DRA and simulated radiation efficiency.

Figure 12 illustrates the simulated and measured radiation patterns of the proposed DRA in *xz*-plane ($\phi = 0^\circ$) and *yz*-plane ($\phi = 90^\circ$). It can be seen that the proposed DRA’s radiation patterns remain stable within the desired operating band. It can be observed from the figure that the difference between LHCP and RHCP radiation levels is more than 18 dB confirming the purity of the LHCP radiation. Figure 13 shows simulated radiation efficiency along with the measured and simulated boresight gain of the proposed CP-DRA versus frequency. It is observed that a squint-free radiation pattern is obtained with a peak gain at boresight compensating the beam squint of $41^\circ$. The antenna efficiency remains at more than $97\%$ throughout the passband of the DRA. Furthermore, the proposed antenna provides a gain of more than $2.85 \, {\text{dBic}}$ within the desired 3-dB AR bandwidth with a peak gain of about $4.64 \, {\text{dBic}}$.

### Comparison with the state-of-the-art designs

Table 1 presents a comparison between the proposed CP-DRA and the previously reported designs, where $\lambda _0$ is the wavelength at the center frequency of the passband in free space. Compared to the state-of-the-art techniques reported in the literature, the proposed antenna offers a relatively wider AR bandwidth with squint-free radiations and a competitive trade-off between compactness and CP bandwidth.

**Table 1:** Comparison with CP DRAs in the literature.

Ref.	$\varepsilon _r$	Antenna dimensions ($\lambda _0^3$)	Imp. BW (%)	AR BW (%)	Peak gain (*dBic*)
^15^	Anisotropic DR	$1.06 \times 1.06 \times 0.123$	33.5	26.3	8.5
^16^	10	$0.50 \times 0.44 \times 0.42$	69.66	44.73	6.34
^19^	10	$0.647 \times 0.647 \times 0.122$	50.8	36	6
^20^	12	$0.575 \times 0.575 \times 0.092$	30.4	25.5	4.95
^21^	10	$4.9 \times 4.9 \times 0.365$	27.7	20	6.8
^22^	10	$0.724 \times 0.724 \times 0.189$	46.9	46.9	4.73
^23^	10	$0.437 \times 0.437 \times 0.336$	59.8	20.8	4.91
^24^	12.8	$1.455 \times 1.455 \times 0.105$	49.7	41	1.5
^25^	9.8	$0.61 \times 0.61 \times 0.273$	30.4	24.6	5.5
This work	10	${0.484} \times {0.484} \times {0.237}$	66.8	54.8	4.64

## Conclusion

A novel wideband circularly polarized DRA with squint-free radiation characteristics has been proposed. The proposed CP-DRA consists of an S-shaped dielectric resonator block with one metalized edge connected to a coaxial probe, two horizontally and vertically loaded rectangular DRs, and a small metal strip attached to the vertical DR connected to the coaxial probe. Additionally, a vertical metal strip is placed parallel to the vertical DR’s outer edge to achieve better antenna bandwidth. To enhance the CP bandwidth, the rectangular DR is modified to form an S-shaped DR by deducting two equal rectangular-shaped blocks from RDR. A 100% correction of beam squinting ($\theta = 41^\circ$) with respect to boresight has been obtained by loading two horizontally and vertically positioned RDRs (L-shaped DR). The experimental results have demonstrated that the proposed CP-DRA achieved about 66.8% (3.71–7.4 GHz) of matching bandwidth, which completely covers the 3-dB AR of about 54.8% (3.72–6.53 GHz) demonstrating its potential for various applications, such as compact communications, satellite communications, 5G Wi-Fi, WLAN, and WAP.
